# Healthcare Workers (HCWs)’ Perceptions and Current Practice of Managing Cognitively Impaired Patients with Chronic Obstructive Pulmonary Disease (COPD)

**DOI:** 10.3390/medicina61010059

**Published:** 2025-01-02

**Authors:** Rayan A. Siraj

**Affiliations:** Department of Respiratory Care, College of Applied Medical Sciences, King Faisal University, Al-Ahasa 31982, Saudi Arabia; rsiraj@kfu.edu.sa

**Keywords:** COPD, cognitive impairment, dementia

## Abstract

*Background and Objectives*: Despite the significant impacts of cognitive impairment on patients with chronic obstructive pulmonary disease (COPD), there is limited information available on healthcare workers’ (HCWs) perceptions, current practice, and barriers to managing COPD patients with cognitive impairment. *Materials and Methods*: A cross-sectional questionnaire was distributed to HCWs in Saudi Arabia between April and December 2023. The collected responses were analysed using descriptive statistics and logistic regression models. *Results*: A total of 890 participants, including nursing, physical, and respiratory therapists, completed the online questionnaire. Over two-thirds of the study participants indicated not having sufficient knowledge or adequate training in managing cognitive impairment in patients with COPD. The majority of HCWs perceive cognitive impairment to underdiagnose COPD (83%), underestimate COPD severity (81%), exacerbate COPD symptoms (80%), and interfere with self-management (81%) and pulmonary rehabilitation (81%). However, less than 45% (*n* = 394) reported having the potential to recognise signs of cognitive impairment during patient encounters. Logistic regression analysis revealed that male healthcare practitioners were more likely to recognise cognitive impairment than females (OR: 1.48; 95% CI: 1.13 to 1.95; *p* < 0.001). Physical and respiratory therapists were more likely to identify cognitive impairment compared to nurses. Additionally, having more years of experience (≥10 years, OR: 1.63; 95% CI: 1.02 to 2.61; *p* = 0.001) and adequate knowledge of cognitive impairment (OR: 6.23; 95% CI: 4.18 to 9.29; *p* = 0.001) were strongly associated with better recognition. Confidence in managing cognitively impaired COPD patients was low, attributed to poor training (64%), inadequate knowledge (64%), and the absence of standardised procedures (58%). *Conclusions*: HCWs agreed upon the negative impacts associated with cognitive impairment in patients with COPD. However, the potential of recognising signs of cognitive impairment and confidently dealing with the existence of cognitive impairment in COPD is not optimal, owing to poor training and limited knowledge. A focus should be given to managing comorbidities alongside COPD.

## 1. Introduction

Although chronic obstructive pulmonary disease (COPD) is widely known as lung disease, it is nevertheless more commonly associated with other comorbid conditions, such as cognitive impairment [1]. There is substantial evidence showing that cognitive impairment in patients with COPD is linked to severe clinical outcome consequences such as increased healthcare usage [2], insufficient medication adherence [3], low pulmonary rehabilitation (PR) uptake [4], and even increased risk of death in some cases [5]. Despite this, cognitive impairment is given little to no consideration during patients’ encounters, leading to being under or even undiagnosed in a significant proportion of patients.

Studies investigating the prevalence of cognitive impairment in COPD have mainly been heterogeneous. Still, current evidence shows that it ranges from 4% to 61% [6,7], depending on the sample size, assessment methods and study designs. In addition, a large matched-cohort study conducted among the UK population showed that patients with COPD are more likely to develop cognitive impairment and subsequent dementia compared to people without COPD [8]. The prevalence and incidence of COPD have been steadily increasing over the last several decades (1990–2019) [9], leading to a corresponding increase in the incidence of COPD-related comorbidities, such as cognitive impairment. However, no studies have attempted to quantify this trend in the Saudi Arabian population. Moreover, cognitive impairment, even at a milder stage, is considered a significant risk of developing irreversible dementia [10]. With the increased prevalence and incidence of cognitive impairment (and its associated impacts on patients with COPD), it is therefore vital to actively detect and manage the presence of cognitive deficits.

It is increasingly known that a proper management intervention starts with timely detection. Indeed, recognising the issue early in its course allows healthcare professionals to intervene and prevent or at least minimise further deterioration in cognitive status. Failure to establish a diagnosis could lead to a missed opportunity to implement appropriate intervention strategies. Because of this, healthcare providers caring for patients with COPD on a daily basis serve as an essential point to detect any changes in cognitive status.

There have yet to be studies conducted on the perceptions and current practices of healthcare workers (HCWs) on detecting and managing cognitive impairment in patients with COPD. Therefore, this study aims to examine healthcare workers’ perceptions of cognitive impairment in COPD patients and investigate the current practices, confidence levels, and barriers to managing cognitive impairment in COPD patients.

## 2. Methods

### 2.1. Study Design and Participants

Between April and December 2023, an online (Survey Monkey) cross-sectional questionnaire was distributed to assess HCWs’ perception and current practice of managing cognitively impaired patients with COPD.

### 2.2. The Questionnaire

This study distributed a five-section online questionnaire consisting of closed-ended elements. The questionnaire was adopted from a previously published one, which was used to assess the attitudes, current practices, and barriers to managing depression in patients with COPD [11]. The questionnaire has gone through several stages to ensure it is aligned with the purpose of this study. Experts with experience managing patients with COPD and cognitive impairment conducted the structure, formulation, and validation. To ensure content and face validity were fulfilled, pilot testing included 20 HCWs from different disciplines (nurses, physical therapists (PTs), and respiratory therapists (RTs)). Those included in the pilot group were excluded from the original study.

The first section of the questionnaire included data related to demographics such as gender, profession (nurses, PTs, and RTs), years of clinical experience, monthly encounters with COPD patients, and whether a practitioner has sufficient knowledge about cognitive impairment or ever received training for caring for patients with cognitive impairment. The second section of the questionnaire includes ten statements that aim to assess the perceptions of HCWs towards the impacts of cognitive impairment in patients with COPD, ranging from 1 (strongly disagreed) to 5 (strongly agreed). The third part of the questionnaire aimed to assess the current practice of HCWs when dealing with COPD patients and cognitive impairment. In this section, HCWs were asked whether they seek to recognise possible cognitive impairment during patients’ encounters and what actions they are more likely to take when detecting and managing cognitive impairment. Section five included six statements which assessed the confidence level when dealing with patients with COPD who have cognitive impairment. A 5-point Likert scale was used, ranging from 1 (not confident) to 5 (completely confident). The final section asked about the perceived barriers to detecting cognitive impairment in patients with COPD.

### 2.3. Sampling Strategy

The target populations were healthcare practitioners encountering patients with COPD on a daily basis. This included nurses, PTs, and RTs. Using a convenience sampling approach, participants were recruited from all regions across the Saudi Kingdom. Participants were reached out via professional Saudi committees to distribute the questionnaire. Social media networks such as Twitter (X), WhatsApp, and Telegram were also used to help reach a greater population.

The aim of the study and the principal investigator’s (PI) identity were provided to the participants before filling out the questionnaire. Participants were informed that no personal information would be collected and their participation was voluntary. It was also clear that the collected data would be used for research purposes and kept confidential. Informed consent was obtained by asking whether participants agreed to participate in the study. By answering “yes”, participants agree to offer anonymous data for the research. The time taken to fill out the questionnaire was between 5 and 7 min.

### 2.4. Ethical Approval

An independent research committee at King Faisal University (ID: KFU-REC-2023-MAR-ETHICS727) has granted ethical approval for this study.

### 2.5. Statistical Analysis

Data management and analyses were performed using STATA version 16.0 software (StataCorp LP, College Station, TX, USA). Only categorical variables were included in this study; thus, results were presented as frequency and percentages. Multivariable logistic regression analyses were conducted to identify factors associated with recognising cognitive impairment in patients with COPD. Independent variables included gender, profession (nurse, physical therapist, respiratory therapist), years of clinical experience, geographical location, and knowledge level. Odds ratios (ORs) with 95% confidence intervals (CIs) were calculated to quantify the strength of associations, and statistical significance was set at *p* < 0.05. Participants with missing or incomplete questionnaires were excluded from the final analyses. Since this was an exploratory study, no sample size calculation was required.

## 3. Results

In total, 890 HCWs filled out the online questionnaire and thus were included in the final analysis. Nurses accounted for 65% of the population, and most participants had 1–4 years of clinical experience (54%), see Table 1. Of significant importance, the vast majority of HCWs indicated not having sufficient knowledge about cognitive impairment in COPD (73%) nor having received adequate training about managing cognitively impaired patients (69%), see Table 1.

### 3.1. The Perceptions of HCWs Towards Cognitive Impairment in Patients with COPD (n = 890)

Most HCWs agreed that cognitive impairment may result from COPD (70%, including 30.21% agree and 39.79% strongly agree), see Table 2. Additionally, 83.04% believed it could lead to underdiagnosis, and 81.24% felt it could result in misdiagnosis of COPD severity. A significant majority recognised its impact on poor medication adherence (81.5%), reduced pulmonary rehabilitation participation (80.74%), exacerbation of symptoms (80%), and impaired self-management (81%), Table 2.

### 3.2. Current Practices and Confidence Level for Detecting and Managing Cognitive Impairment in Patients with COPD (n = 890)

Out of 890 HCWs, 45% (*n* = 394) reported aiming to identify cognitive impairment during patient encounters. Most relied on psychiatric symptoms (84.5%), family history of cognitive impairment (76.4%), and memory deficits (63.7%) as criteria. For interventions, 83% provided information on the link between COPD and cognitive impairment, while 82% and 73% discussed referrals to mental health clinics and pulmonary rehabilitation, respectively, see Table 3.

Factors associated with identifying cognitive impairment in patients with COPD were investigated using univariate multivariate and logistic regression analyses. Results revealed that male healthcare practitioners were more likely to recognise signs of cognitive impairment than females (OR: 1.48; 95% CI: 1.13 to 1.95; *p* < 0.001), see Table 4. In addition, physical and respiratory therapists, compared to nurses, were more likely to identify cognitive impairment. Having more years of experience (≥10 years) and adequate knowledge of cognitive impairment was associated with identifying cognitive impairment in COPD patients (OR 1.63; 95% CI: 1.02 to 2.61; *p* = 0.001) and (OR 6.23; 95% CI: 4.18 to 9.29; *p* = 0.001), respectively. Results also remained the same in the multivariate logistic regression analyses.

Of those who indicated the potential to recognise cognitive impairment during patients’ encounters (*n* = 394), the vast majority showed a low level of confidence when responding to behaviour problems, telling a diagnosis to the patients, educating the patients about cognitive impairment in COPD, and knowing which signs to look for to detect the presence of cognitive impairment, see Table 5.

### 3.3. HCWs’ Perception of the Barriers to Cognitive Impairment Detection and Management in Patients with COPD

**Figure 1:**
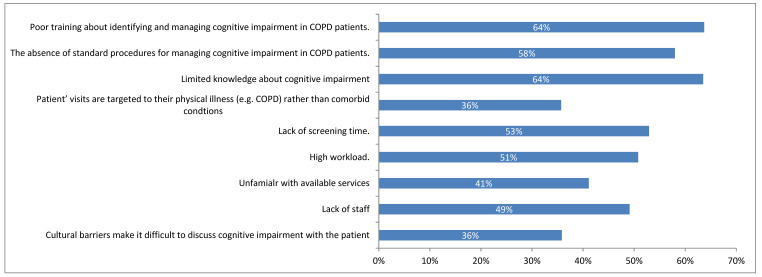
Perceived barriers associated with identifying and managing cognitive impairment in patients with COPD (*n* = 890).

As shown in Figure 1, the most common barriers to detecting and managing cognitive impairment in patients with COPD were as follows: poor training (64%), limited knowledge (64%), absence of standardised procedure (58%), and lack of screen time (53%).

## 4. Discussion

In this study, we aimed to assess the perceptions of healthcare workers from different specialities towards managing patients with COPD and cognitive impairment. This study’s findings revealed that HCWs perceive the negative impacts of cognitive impairment on COPD, such as underdiagnosing COPD, underestimating COPD severity, interfering with self-management, poor adherence to medication regimens, and increased risk of exacerbation. Despite this, only 45% of this study’s participants aim to recognise signs of cognitive impairment during patients’ encounters. This might be justified by the lack of confidence when caring for COPD patients who suffer from cognitive impairment. Participants revealed that poor training and limited knowledge hindered their practice.

Increasing evidence shows that cognitive impairment is among the common comorbidities associated with COPD, with severe impacts on clinical management [6,7,12,13]. This includes underdiagnosis of COPD [14], improper inhaler techniques [3], and poor adherence to medication regimens and pulmonary rehabilitation programs [3,4]. Although HCWs, overall, agreed upon these consequences associated with cognitive impairment, they do not see value in routine screening. Indeed, despite its controversial role, screening could help determine which patients need further cognitive assessment and are, therefore, provided with optimal care. If cognitive impairment remains un- or under-diagnosed, there is a potential risk that cognitive impairment may progress to irreversible dementia [10]. For this reason, HWCs caring for patients with COPD should keep in mind the existence of cognitive symptoms during patients’ encounters.

Healthcare workers dealing with patients with COPD on a daily basis have an essential role in identifying cognitive impairment signs during patient encounters. To explain, patients might not adhere to a proper inhaler technique (as it requires planning and sequencing), which could be compromised in case of cognitive impairment [3], leading to poor drug delivery and, thus, patients’ deterioration. However, if HCWs actively seek to identify such signs, this issue might be minimised or even prevented. Indeed, early identification of cognitive impairment allows for appropriate interventions and, thus, better clinical outcomes. It is nevertheless important to mention that the proper use of inhalers is a crucial component of effective self-management [15], which has been linked to better clinical outcomes.

Cognitive impairment is common among patients with COPD, and it is also associated with patients’ severity [13]. However, it is not being screened or assessed during patients’ encounters. This study shows that less than half of the participants aim to identify potential cognitive impairment. This could be due to a lack of training towards knowing the signs and symptoms of cognitive impairment or even managing patients with COPD with comorbidities, one of which is cognitive impairment. Indeed, current guidelines do not provide specific recommendations on managing COPD comorbidities despite emphasising recognising them. In addition, HCWs (such as nurses, PTs, and RTs) might only focus on providing healthcare services (pharmacological and non-pharmacological interventions) that target COPD symptoms instead of other existing conditions, ultimately leading to low detection of cognitive impairment.

The impacts of cognitive impairment on patients with COPD are significant, leading to worse clinical outcomes. Therefore, actions, such as managing modifiable risk factors (e.g., smoking, hypoxia, and cardiovascular comorbidities), which have been shown to increase the risk of cognitive impairment in patients with COPD [16,17], should all be considered during patients’ encounters. In this study, most participants indicated that they would educate the patients about the link between cognitive impairment and COPD, as well as discuss a referral to a mental health clinic. It is also essential to emphasise the role of pulmonary rehabilitation programs in improving cognitive function in COPD patients. Indeed, Pereira et al. showed that a 3-month program was associated with a significant improvement in the cognitive function of COPD patients, highlighting the beneficial impacts on PR in this population [18].

It is acknowledged that caring for patients with cognitive impairment is challenging, even among highly qualified healthcare practitioners. Previous data showed that primary care physicians have low confidence when it comes to managing patients with cognitive impairment. In line with the current literature, our findings showed that HCWs have an overall low level of confidence when caring for patients with COPD and cognitive impairment [19,20]. It is worth mentioning, however, that managing COPD by itself is challenging and becomes even more problematic when it co-exists with other comorbidities, one of which is cognitive impairment [21,22]. While COPD guidelines encourage identifying comorbidities, they do not provide specific guidance on how to do so. Therefore, national and international guidelines should provide specific guidance on recognising and managing comorbidities in COPD.

Barriers such as lack of knowledge, poor training, and the absence of specific recommendations for managing comorbidities in COPD (e.g., cognitive impairment) not only reduce healthcare workers’ confidence but also contribute to suboptimal patient management. These systemic challenges hinder the effective detection and management of cognitive impairment, potentially leading to missed or incorrect diagnoses, inadequate patient care, and poorer health outcomes.

To address these issues, the healthcare system must provide more comprehensive education and training programs focusing on recognising and managing comorbidities in COPD patients. Developing standardised clinical guidelines and protocols will also improve HCWs’ ability to manage these conditions effectively. By investing in these areas, the healthcare system can enhance HCWs’ competence and confidence, resulting in earlier detection of cognitive impairment and more appropriate interventions, ultimately leading to improved clinical outcomes for patients with COPD.

## 5. Study Implications

This study’s findings have significant implications for improving clinical practice, especially in the early recognition of cognitive impairment in COPD patients. Although healthcare workers (HCWs) do not have the authority to diagnose cognitive impairment, their role in recognising early signs and symptoms is crucial. Cognitive impairment in COPD patients often goes undetected, which can lead to worse clinical outcomes, including poor treatment adherence, decreased self-management, and even accelerated cognitive decline. HCWs, being the frontline of patient care, are in an ideal position to observe subtle cognitive changes during routine interactions. By recognising these signs early and promptly informing physicians, HCWs can facilitate timely referrals for further cognitive assessments and appropriate interventions. While this requires intensive training in knowing the signs and symptoms of cognitive impairment, this early recognition could enable physicians to diagnose cognitive impairment at an earlier stage, potentially preventing its progression to more severe conditions like dementia [1,23]. Therefore, providing HCWs with the necessary training and tools to detect cognitive impairment symptoms and developing clear protocols for when to escalate concerns to medical professionals can significantly enhance the quality of care and lead to better patient outcomes in managing COPD and its cognitive comorbidities.

## 6. Study Limitations

Initially, the cross-sectional nature of this study’s design precludes the investigation of a cause-and-effect relationship. Instead, it only offers a brief overview of the current practices of managing cognitive impairment in patients with COPD without considering the changes over time. While this design was appropriate for capturing current practices, a longitudinal approach would provide deeper insights into how interventions and practices evolve and their long-term impact on patient outcomes. Further, despite the potential for selection bias to be introduced by the method of selection employed in this study, participants were recruited from all over the country, thus offering high external validity. Therefore, the likelihood of introducing methodological flaws is reduced, as clinicians included in this study were from various geographical locations in Saudi Arabia. Although self-reported questionnaires may be cost-effective and time-efficient, they may introduce self-reporting bias, as clinicians may overestimate their confidence levels or practices. Of clinical importance, though, this study sheds some light on a significant practice gap in recognising and managing cognitive impairment in COPD patients, highlighting the need for more education and training for HCWs caring for COPD patients.

## 7. Conclusions

Overall, HCWs agreed upon the negative impacts of cognitive impairment on patients with COPD. However, the prevalence of recognising signs of cognitive impairment and confidently managing them is relatively low, attributed to poor knowledge and skills and the absence of specific management recommendations. Interventions focusing on educating and training HCWs on COPD comorbidities in general and cognitive impairment in particular should, therefore, be applied.

## Figures and Tables

**Table 1:** Demographics and professional background of the study respondents (*n* = 890).

Demographics	Frequency (%)
Gender	
Male	564 (64.37%)
Female	326 (36.63%)
Geographic Location	
Central Region	98 (11.01%)
Eastern Region	130 (14.61%)
Western Region	96 (10.79%)
Northern Region	158 (17.75%)
Southern Region	408 (45.84%)
Profession	
Nurse	581 (65.28%)
Physical therapist	58 (6.51%)
Respiratory therapist	251 (28.21%)
Primary place of work	
Governmental hospital	783 (88%)
Private hospital	107 (12%)
The average number of COPD patients seen per month	
≤10	512 (57.53%)
11–20	240 (26.97)
≥21	138 (15.51%)
Years of clinical experience with COPD patients	
<1 year	101 (11.35%)
1–4 years	480 (53.93%)
5–9 years	251 (28.20%)
≥10 years	58 (6.51%)
Have sufficient knowledge about cognitive impairment in people with COPD	
Yes	249 (27%)
No	640 (73%)
Received specific training for managing cognitive impairment	
Yes	276 (31%)
No	614 (69%)

Data are presented as frequencies and percentages. COPD: chronic obstructive pulmonary disease.

**Table 2:** HCWs’ perceptions about cognitive impairment in patients with COPD (*n* = 890).

Item	Frequency (%)
Cognitive impairment may develop as a result of impaired lung function, such as COPD.	
Strongly disagree	13 (1.46%)
Disagree	37 (4.16%)
Neither agree nor disagree	217 (24.38%)
Agree	349 (39.21%)
Strongly agree	274 (30.79%)
Cognitive impairment may lead to underdiagnoses of COPD.	
Strongly disagree	6 (0.67%)
Disagree	20 (2.25%)
Neither agree nor disagree	125 (14.04%)
Agree	435 (48.88%)
Strongly agree	304 (34.16%)
Cognitive impairment may lead to a misdiagnosis of the COPD severity	
Strongly disagree	6 (0.67%)
Disagree	22 (2.47%)
Neither agree nor disagree	139 (15.62%)
Agree	377 (42.36%)
Strongly agree	364 (38.88%)
Cognitive impairment is responsible for poor adherence to medications in patients with COPD.	
Strongly disagree	7 (0.79%)
Disagree	27 (3.03%)
Neither agree nor disagree	131 (14.72%)
Agree	398 (44.72%)
Strongly agree	327 (36.74%)
Cognitive impairment is responsible for insufficient adherence to pulmonary rehabilitation in patients with COPD.	
Strongly disagree	6 (0.67%)
Disagree	24 (2.70%)
Neither agree nor disagree	143 (16.07%)
Agree	428 (48.09%)
Strongly agree	289 (32.74%)
Cognitive impairment may lead to underestimation of exercise performance in patients with COPD	
Strongly disagree	6 (0.67%)
Disagree	20 (0.25%)
Neither agree nor disagree	159 (17.91%)
Agree	394 (44.37%)
Strongly agree	309 (34.80%)
Cognitive impairment may lead to treatment refusal in patients with COPD	
Strongly disagree	4 (0.45%)
Disagree	20 (2.25%)
Neither agree nor disagree	134 (15.06%)
Agree	368 (41.35%)
Strongly agree	364 (40.90%)
There is little value in routinely screening for cognitive impairment in patients with COPD.	
Strongly disagree	11 (1.24%)
Disagree	22 (2.48%)
Neither agree nor disagree	149 (16.78%)
Agree	372 (41.89%)
Strongly agree	334 (37.61%)
Cognitive impairment exacerbates the symptoms of COPD.	
Strongly disagree	7 (0.79%)
Disagree	21 (2.36%)
Neither agree nor disagree	145 (16.29%)
Agree	398 (44.72%)
Strongly agree	319 (35.84%)
Cognitive impairment impairs patient self-management of COPD	
Strongly disagree	8 (0.90%)
Disagree	21 (2.36%)
Neither agree nor disagree	133 (14.94%)
Agree	407 (45.73%)
Strongly agree	321 (36.07%)

Data are presented as frequencies and percentages. COPD: chronic obstructive pulmonary disease.

**Table 3:** Current practice in encountering COPD patients.

Item	Frequency (%)
Do you aim to recognise possible cognitive impairment during patients with COPD encounters?	
Yes	394 (44.26%)
No	496 (55.73%)
When aiming to detect and manage cognitive impairment in patients with COPD, what actions are you more likely to take?	
I take memory deficit as a criterion for cognitive impairment detection.	251 (63.70%)
I take psychiatric symptoms as the criteria for cognitive impairment detection.	333 (84.51%)
I would ask if the patient has a family history of Alzheimer’s disease	301 (76.39%)
I would detect risk factors.	179 (45.43%)
I would provide information about cognitive impairment.	330 (83.76%)
I would discuss a referral to a mental health clinic.	322 (81.73%)
I would discuss a referral to pulmonary rehabilitation.	288 (73.10%)
I would discuss a referral to a neurologist.	122 (30.96%)
I would suggest supplemental oxygen therapy.	283 (71.83%)

Data are presented as frequencies and percentages. COPD: chronic obstructive pulmonary disease.

**Table 4:** Bivariate and multivariate logistic regression models of the factors associated with identifying cognitive impairment in patients with COPD.

Descriptor	OR (95% CI)	AOR (95% CI)	*p* Value
Sex			
Female	1	1	
Male	1.48 (1.13 to 1.95)	2.06 (1.52 to 2.08)	<0.001
Specialities			
Nurse	1	1	
Physical therapy	3.57 (1.97 to 6.47)	7.21 (3.73 to 13.90)	<0.001
Respiratory therapy	1.81 (1.34 to 2.44)	1.84 (1.34 to 2.55)	<0.001
Geographical location			
Central Region	1	1	
Eastern Region	2.14 (1.20 to 3.79)	2.13 (1.16 to 3.91)	0.014
Northern Region	7.33 (4.14 to 12.97)	7.34 (3.95 to 13.63)	<0.001
Southern Region	1.10 (0.66 to 1.82)	1.25 (0.73 to 2.14)	0.403
Western Region	2.57 (1.40 to 4.72)	2.21 (1.15 to 4.24)	0.016
Years of experience			
≤1 year	1	1	
1–4 years	1.12 (0.72 to 1.74)	1.41 (0.76 to 2.32)	0.172
5–9 years	1.63 (0.85 to 3.13)	1.79 (0.87 to 3.66)	0.11
≥10 years	1.63 (1.02 to 2.61)	1.72 (1.01 to 2.93)	0.047
Have sufficient knowledge			
No	1	1	
Yes	6.23 (4.18 to 9.29)	3.91 (2.88 to 5.31)	<0.001
Place of work			
Governmental Hospital	1	1	
Private Hospital	0.60 (0.39 to 0.91)	0.53 (0.32 to 0.88)	0.016

Abbreviation: CI: confidence interval; OR: odds ratio; AOR: adjusted odds ratio.

**Table 5:** Confidence in caring for patients with COPD and cognitive impairment (*n* = 394).

Item	Frequency (%)
In responding to co-existing behaviour problems in patients with COPD, I feel	
Completely confident	12 (3.05%)
Fairly confident	43 (0.76%)
Somewhat confident	154 (39.09%)
Slightly confident	104 (26.40%)
Not confident	121 (30.71%)
In telling the patients the diagnosis, I feel	
Completely confident	11 (2.79%)
Fairly confident	5 (1.27%)
Somewhat confident	69 (17.51%)
Slightly confident	139 (35.28%)
Not confident	170 (43.15%)
In discussing concerns about possible cognitive impairment with a patient’s family members, I feel	
Completely confident	12 (3.05%)
Fairly confident	2 (0.51%)
Somewhat confident	92 (23.35%)
Slightly confident	155 (39.34%)
Not confident	133 (33.76%)
In knowing which signs to look for to tell if a patient with COPD might be cognitively impaired, I feel	
Completely confident	12 (3.05%)
Fairly confident	4 (1.02%)
Somewhat confident	84 (21.32%)
Slightly confident	162 (41.12%)
Not confident	132 (33.50%)
In providing education on the link between COPD and cognitive impairment, I feel	
Completely confident	9 (2.28%)
Fairly confident	8 (2.03%)
Somewhat confident	85 (21.57%)
Slightly confident	152 (38.58%)
Not confident	140 (35.53%)
In directing a patient who might be cognitively impaired to appropriate services or agencies, I feel	
Completely confident	8 (2.03%)
Fairly confident	7 (1.78%)
Somewhat confident	78 (19.80%)
Slightly confident	162 (41.12%)
Not confident	139 (35.28%)

Data are presented as frequencies and percentages. COPD: chronic obstructive pulmonary disease.

## Data Availability

All relevant data are within the paper.

## Abbreviations

BMI	Body Mass Index
CI	Confidence Interval
COPD	Chronic Obstructive Pulmonary Disease
HCWs	Healthcare Workers
OR	Odds Ratio
PR	Pulmonary Rehabilitation
PTs	Physical Therapists
RTs	Respiratory Therapists
STATA	Statistical Software by StataCorp
UK	United Kingdom
